# Network of doctors for multimorbidity and diabetes — the NOMAD intervention: protocol for feasibility trial of multidisciplinary team conferences for people with diabetes and multimorbidity

**DOI:** 10.1186/s40814-024-01517-0

**Published:** 2024-06-15

**Authors:** Stine Jorstad Bugge, Daniel Pilsgaard Henriksen, Per Damkier, Martin Torp Rahbek, Karoline Schousboe, Mette Juel Rothmann, Marianne Kjær Poulsen, Camilla Hansen, Subagini Nagarajah, Per Bruno Jensen, Sofie Lock Johansson, Vasiliki Panou, Ida Ransby Schneider, Charlotte Gjørup Pedersen, Jonas Dahl Andersen, Jørgen Hangaard, Ann-Dorthe Olsen Zwisler

**Affiliations:** 1 Steno Diabetes Centre Odense, Odense University Hospital, 5000 Odense, Denmark; 2 Department of Pharmacology, Odense University Hospital, Odense, Denmark; 3 Department of Clinical Research, University of Southern Denmark, Odense, Denmark; 4 Department of Public Health, University of Southern Denmark, Odense, Denmark; 5 Department of Nephrology, Odense University Hospital, Odense, Denmark; 6 Department of Respiratory Medicine, Odense University Hospital, Odense, Denmark; 7 Department of Cardiology, Odense University Hospital, Odense, Denmark; 8 Steno Diabetes Centre Aarhus, Aarhus University Hospital, Aarhus, Denmark; 9 Department of Public Health, Aarhus University, Aarhus, Denmark; 10 Steno Diabetes Centre Northern Jutland, Aalborg University Hospital, Aalborg, Denmark; 11 Department of Health Science and Technology, Aalborg University, Aalborg, Denmark; 12 REHPA, The Danish Knowledge Centre for Rehabilitation and Palliative Care, Odense University Hospital, Nyborg, Denmark

**Keywords:** Feasibility trial, Diabetes, Multimorbidity, Comorbidity, Multidisciplinary team, Complex intervention, Patient-reported outcome, Process evaluation

## Abstract

**Background:**

The prevalence of diabetes and coexisting multimorbidity rises worldwide. Treatment of this patient group can be complex. Providing an evidence-based, coherent, and patient-centred treatment of patients with multimorbidity poses a challenge in healthcare systems, which are typically designed to deliver disease-specific care. We propose an intervention comprising multidisciplinary team conferences (MDTs) to address this issue. The MDT consists of medical specialists in five different specialities meeting to discuss multimorbid diabetes patients. This protocol describes a feasibility test of MDTs designed to coordinate care and improve quality of life for people with diabetes and multimorbidity.

**Methods:**

A mixed-methods one-arm feasibility test of the MDT. Feasibility will be assessed through prospectively collected data. We will explore patient perspectives through patient-reported outcomes (PROs) and assess the feasibility of electronic questionnaires. Feasibility outcomes are recruitment, PRO completion, technical difficulties, impact of MDT, and doctor preparation time. During 17 months, up to 112 participants will be recruited. We will report results narratively and by the use of descriptive statistics. The collected data will form the basis for a future large-scale randomised trial.

**Discussion:**

A multidisciplinary approach focusing on better management of diabetic patients suffering from multimorbidity may improve functional status, quality of life, and health outcomes. Multimorbidity and diabetes are highly prevalent in our healthcare system, but we lack a solid evidence-based approach to patient-centred care for these patients. This study represents the initial steps towards building such evidence. The concept can be efficiency tested in a randomised setting, if found feasible to intervention providers and receivers. If not, we will have gained experience on how to manage diabetes and multimorbidity as well as organisational aspects, which together may generate hypotheses for research on how to handle multimorbidity in the future.

**Administrative information:**

Protocol version: 01

**Trial registration:**

NCT05913726 — registration date: 21 June 2023

**Supplementary Information:**

The online version contains supplementary material available at 10.1186/s40814-024-01517-0.

## Background

WHO defines multimorbidity as the coexistence of two or more chronic diseases in the same person [1]. Due to increasing life expectancy, an ever-increasing number of people struggle with chronic conditions and complex multimorbidity [2]. Diabetes is one of the four major groups of noncommunicable diseases as defined by the WHO, with a global prevalence in 2019 estimated to 463 million people [3]. The same year, diabetes caused two million premature deaths worldwide [4]. Diabetes is associated with several other comorbidities, such as cardiovascular diseases, mental health disorders, and cancer [5–7]. In Australia, researchers report that 90% of people with type 2 diabetes also have another chronic condition [8], and a Canadian study found that nearly 40% of people with type 2 diabetes had two or more concurrent chronic diseases [9]. A systematic review reported that one of the most frequent combinations of chronic conditions is that of cardiometabolic conditions such as high blood pressure, diabetes, obesity, and ischaemic heart disease [10]. In people with type 2 diabetes, the all-cause mortality rises with increasing number of chronic conditions [11].

Having multiple chronic conditions may often entail clinical check-ups at multiple departments and seeing many different health care providers. Coordinating treatment and collecting prescription drugs can be a challenge for these patients. A lack of coordination may confuse and mentally exhaust the patient, decrease adherence and compliance, and, ultimately, reduce life quality and expectancy [12–14]. The patient may travel like a *nomad* between departments, specialists, and across healthcare sectors, possibly left uncertain of whom to contact if problems arise.

Multidisciplinary team conferences (MDT) are utilised in many parts of the healthcare system and serve as a well-established tool to aid clinical decision-making [15–17]. MDTs promote coordination and coherence in patient care and management [18, 19]. They can tailor care and management to suit the individual patient with complex needs by bringing together expertise from different medical specialties and healthcare professionals. Historically, the MDT approach resides in oncological context where the complexity of diagnostics and treatments is high. This approach has been reported beneficial, yet researchers encourage further research in the impact of MDT on quality of life and in further strategies to incorporate patient perspectives in the MDTs [15, 19].

In recent years, the MDT approach has expanded to non-oncological fields. A recent scoping review on the topic of physician-led in-hospital MDTs in chronic non-malignant diseases concluded that MDT care for patients with multimorbidity may positively affect the treatment, but the literature is scarce [20]. Another recent review reports that MDT for people with diabetes and comorbidities has a positive effect in glycaemic control and mental health outcomes but suggests a more cross-sectorial approach in future studies [21].

Thus, Steno Diabetes Centre Odense (SDCO) has developed a concept of MDT called “Network Of doctors for Multimorbidity And Diabetes — NOMAD” which is ready to be feasibility tested before moving on to a large-scale randomised controlled trial (RCT). We have formed the following hypotheses for the feasibility test:

- The NOMAD intervention can function in a clinical setting and is acceptable to intervention providers and recipients.
- The present concept will be feasible in a future RCT.

Acceptability means that a person finds the intervention appropriate, based on anticipated or experienced cognitive and emotional responses to the intervention [22].

## Methods

### Study design

A mixed-methods one-arm feasibility study with follow-up on persons referred to the NOMAD. The study has an explorative part, looking into the content of the conference discussions and assessing mediators and moderators to feasibility of NOMAD (a process evaluation). This protocol is written in line with CONSORT 2010 statement: extension to randomised pilot and feasibility studies [23] and guidelines for inclusion of patient-reported outcomes in clinical trial protocols, SPIRIT-PRO Extension [24].

### Setting and context

The Danish population amounts to 5.8 million people [25]. Healthcare is free of charge for all citizens in Denmark. The Region of Southern Denmark, where the trial takes place, inhabits 1.2 million people [26] with Odense University Hospital (OUH) as the largest and most specialised hospital. It also serves as local hospital for approximately 275,000 people [27] (catchment area). SDCO introduced the NOMAD as a clinical initiative for all citizens who meet eligibility criteria and who live in OUH catchment area or have general practitioner (GP) on the island of Funen.

### Participants

Adult patients (over 18 years) referred to the NOMAD at OUH from August 2023 until December 2024 (17 months) who have returned written consent to participate. We did several things to ensure recruitment, such as presenting the NOMAD concept at each of the involved departments, producing a pamphlet aimed at doctors and patients, and sending a letter describing the concept as well as paying physical visits to GP. The patient is referred from clinical departments, such as Cardiology, Respiratory Medicine, Nephrology, and/or Diabetology/Steno Diabetes Centre, or from the GP. Referral criteria include patients with any type of diabetes and one or more concurrent chronic conditions within cardiology, respiratory medicine, or nephrology, and complexity in treatment/management, e.g. difficulty in specific treatment, symptoms or other patient complaints, lack of compliance, or polypharmacy.

### Intervention development

**Fig. 1:**
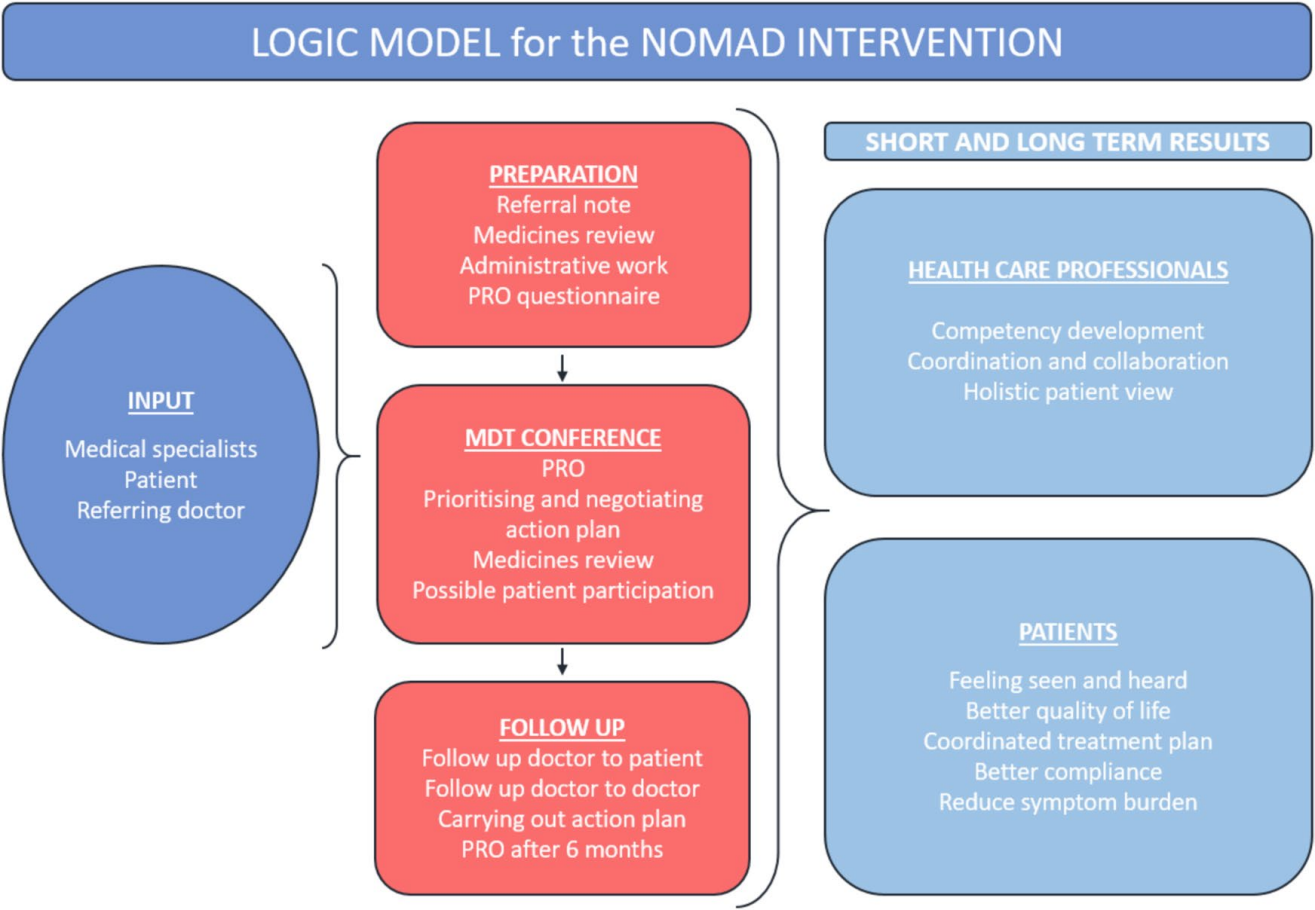
Logic model illustrating the mechanisms of change of the NOMAD intervention. This simplified version of the original logic model focuses on elements that pertain to the feasibility study, and that we address directly or indirectly in the collected data and corresponding outcomes. The input signifies the resources needed to perform the NOMAD intervention. The red boxes indicate elements pertinent to conducting the conference and follow up. The short- and long-term results are divided into results relevant to health care professional and patients respectively. NOMAD, Network of Doctors for Multimorbidity and Diabetes; PRO, patient-reported outcome; MDT, multidisciplinary team conferences

When developing the NOMAD intervention, we followed Medical Research Council’s (MRC) framework for developing and evaluating complex interventions [28] and the “Guidance on how to develop complex interventions to improve health and healthcare” [29]. The development process commenced in 2020, starting with the establishment of dedicated working and research groups. The groups consist of medical specialists within the relevant fields of medicine and researchers with expertise in relevant research areas. When establishing the NOMAD team, factors like local resources and department capacities played a role. For instance, we initially wanted a psychologist in the team, but this was not possible at the time. According to MRC guidelines, we chose a dynamic and iterative approach. An early version of the intervention was pilot-tested in 2021 resulting in a preliminary evaluation report in 2021 (available upon request) with interviews of patients and different healthcare professionals. It showed necessity for a closer collaboration with GP and the need for patient involvement in the decision-making. We refined and adjusted the intervention through working group meetings and workshops. We put great effort in understanding the possible mechanisms of change with construction of a comprehensive logic model. Figure 1 provides a simplified version of the logic model. Further, we conducted a journal audit to gain insights into topics of discussion in the NOMAD conferences. The development process resulted in a final intervention presented in Template for Intervention Description and Replication (TIDieR) format (Appendix 1) and supported by a written, generic manual in national language describing how to set up and perform this type of conferences (available upon request). We published an official local guideline for hospital staff the OUH guidelines collection. The intervention is briefly outlined below.

### Intervention

Every two weeks, a senior registrar/chief physician and a resident doctor from the involved departments (cardiology, diabetes, respiratory medicine, nephrology, and clinical pharmacology) meet to discuss up to four patient cases. The patients are referred to the NOMAD by either one of the involved departments or from their GP. From time of referral to NOMAD conference, usually 1 to 4 weeks pass, allowing the diabetes department (SDCO) to make necessary preparations, such as electronically send out and receive PRO questionnaires. In addition, all participating doctors have protected time to prepare for the conference, which is defined in an agreement between the collaborating departments. Especially, the clinical pharmacologists require several hours preparation time per case to ensure a high quality and systematic review of a patient’s previous hospital contacts and medicines.

If a GP refers, he/she is encouraged to participate in the conference by video link with or without the patient.

A consultant physician from SDCO conducts the conference. The discussion proceeds in a formalised manner, ensuring all doctors get to contribute while keeping a tight schedule. One doctor forms a note of NOMAD conclusions and recommendations based on an oral discussion summary. This note also states who is responsible for following up on treatment.

Six months after the NOMAD conference, we send out the PRO questionnaire again. A healthcare professional reviews the answers and contacts the patient by telephone. This element has a twofold purpose: (1) to follow up on NOMAD recommendations and (2) to discuss their 6-month PRO-questionnaire answers.

### Patient-reported outcome

**Fig. 2:**
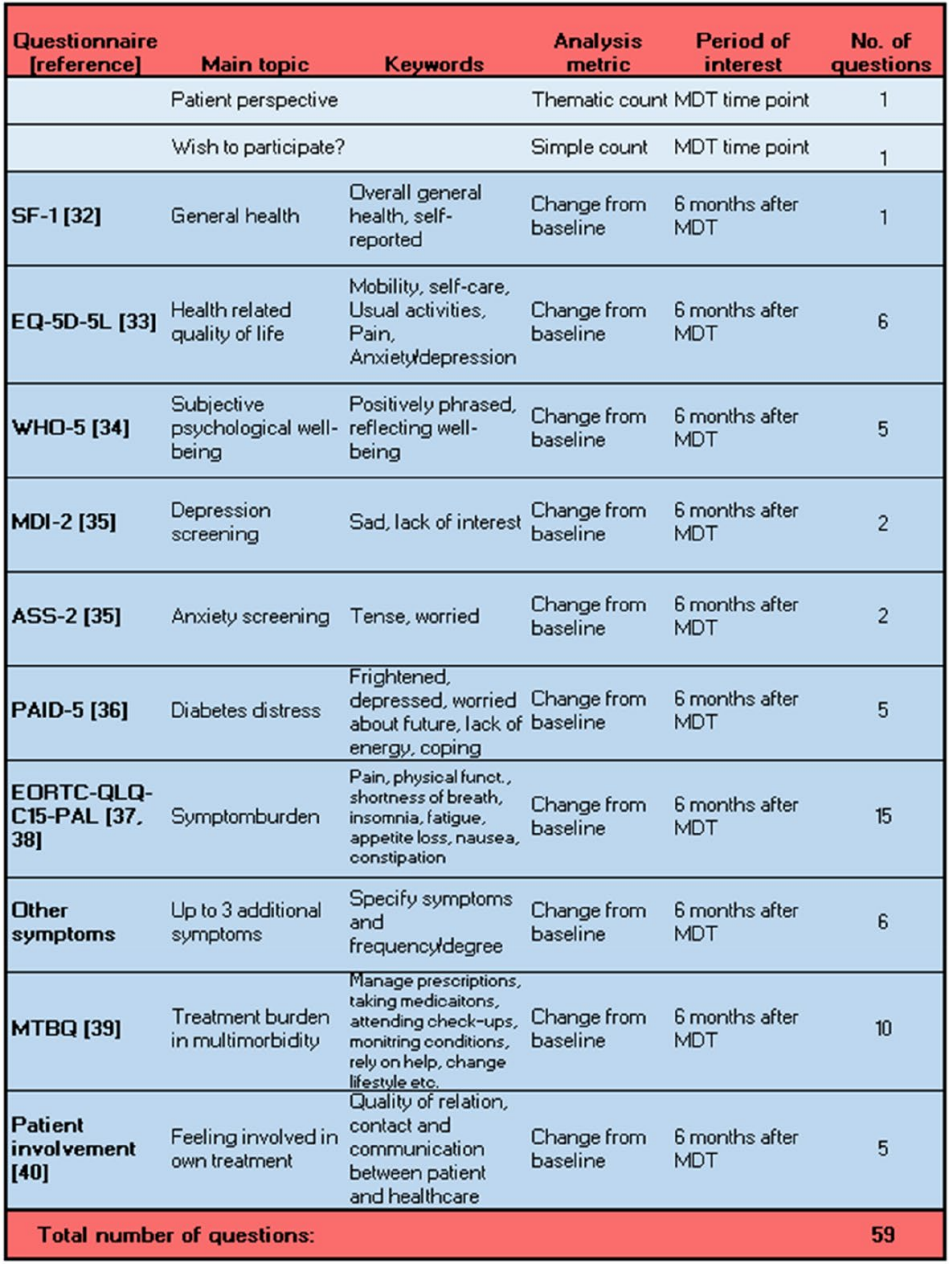
PRO questionnaires

The PRO element is handled according to ethical considerations by Cruz Rivera et al. [30]. The purpose of the questionnaires is to enlighten a multitude of patient life aspects, especially the aspects of living with diabetes and concurrent chronic disease. The PRO questionnaire helps inform NOMAD discussions, as it provides PRO concerning health-related quality of life (HRQoL). A specific PRO group (IDR, CG, JDA, and ADZ) reviewed the literature to gain insights into which validated generic questionnaires would be relevant in diabetes and multimorbidity. Inspired by current literature [31], the context of MDT, and the Danish healthcare system, we chose the following questionnaires: SF1 [32], EQ-5D-5L [33], WHO-5 [34], MDI-2, ASS-2 [35], PAID-5 [36], EORTC-QLQ-C15-PAL [37, 38], and MTBQ [39] and five validated questions about patient involvement [40]. In establishing the PRO domains, we considered the following: (1) validated and recommended questionnaires available in Danish, (2) limiting the number of questions in order to limit the burden of answering, and (3) inclusivity and participant autonomy, when choosing an electronic approach to send out and administer the questionnaires. A complete list of questionnaire selection and description are provided in Fig. 2.

### Progression criteria (see Table 1)

**Table 1:** Progression criteria with traffic light system

**Progression criteria**	**Monitored how**		**Actions**	
		Green	Amber	Red
**Recruitment**	Continuously throughout the project period, simple count of inclusions every 2 weeks	3–4 patients per MDT: continue	2–3 patients per MDT: look into motivational efforts and reminder information to raise referral rates	< 2 patients per MDT: use MDT time slot to make adjustments (e.g. can nurses also refer? Allow patient self-referral?)
**Adherence to protocol**	Every 3 months, assess if there are uncertainties too great to continue (e.g. impossible to keep time schedule in MDT, technical issues like video conference not working)	All or close to all process indicators^a^ are being followed: continue	2–10 process indicators are not being followed: consider revising protocol. If time schedule in MDT is not kept, downscaling to 3 patients per MDT might be necessary	> 10 process indicators are not being followed: consider pausing the project in order to evaluate and redesign intervention
**Data completeness**	Every 6 months, evaluate if the data collection is complete enough. Missing answers in surveys? Unable to retrieve certain register data?	> 80% data completeness: continue	70–80% data completeness: make efforts to collect missing data (e.g. contacting participants)	< 70% data completeness: consider revising protocol. Can data be obtained otherwise? Can data be excluded from study?
**Participant retention**	Every 6 months, register how many participants lost to follow-up. Simple count and register reason if known (e.g. death, unwilling to participate)	Loss to follow-up caused by death, change of mind, or unable to participate will of course be accepted	If not returning questionnaire at 6-month follow-up, then contact by phone to remind and encourage	If < 75% complete follow-up, then look into protocol amendments

^a^ Process indicators are listed in Appendix 2

The number of referred patients is crucial for this study to succeed and to progress into an RCT. Our goal is four patients on each NOMAD conference. We will continuously monitor the rate of referrals in order to take action if needed. Adherence to protocol is monitored according to a list of pre-defined process indicators (Appendix 2). We also monitor data completeness and participant retention.

### Process evaluation

In order to gain further understanding of intervention mechanisms, organisation, and acceptability among end users, we consider a qualitative process evaluation appropriate. By conducting semi-structured interviews with intervention providers, we hope to enlighten mediators and moderators to successful intervention organisation. When selecting individuals for interviews, we will keep a principle of diversity in mind, making sure the interviewees represent both genders, experience levels, medical specialties, and roles in relation to the current project. Interviews will be transcribed verbatim, and we will use a thematic analysis approach to organise and condensate the information [41]. The results from the qualitative process evaluation will be published in a separate paper.

### Variables

We divide variables into two categories: process-related variables and person-related variables. The process-related variables we subdivide according to chronology: before, during, and after the NOMAD conference. Examples of process-related variables are reason for referral, who is referring and does the MDT keep time schedule. We subdivide the person-related variables into clinical (e.g. blood pressure, HbA1C, creatinine), PROs, and organisational variables (number of hospital contacts, where diabetes is treated, number of active treatment courses, physician learning). A full list of variables is provided in Appendix 3 (Figure 3b).

### Outcomes

Primary outcomes will enlighten feasibility. These include recruitment, proportion of completed PRO-questionnaires, amount of technical difficulties in relation to the conference, count of how often discussions exceed 30 min, and how many minutes the clinicians take to prepare for MDT. Secondary outcomes will elucidate possible effects of the NOMAD intervention and include clinical effects for the patient and degrees of clinician self-reported learning.

### Data collection and management

We will collect data continuously throughout the project period: at the time of referral, the time of the NOMAD conference, and at 6-month follow-up. Appendix 3 provides an overview of data collection flow (Figure 4a). The data will come from a combination of questionnaires, registers, and electronic patient records. Data concerning referral, NOMAD discussions, and recommendations completed PRO questionnaires, and basic clinical information will be collected from electronic patient records. Demographic data and healthcare utilisation will be extracted from national registers as well as information on how many and which diagnoses each patient has. Through a pre-study journal audit, we established a coding system to register the NOMAD discussion components. We elaborate on the coding system in Appendix 4. At 6-month follow-up, we will collect data concerning diagnoses, laboratory values, medicines, and hospital contacts. For each patient case discussed on the NOMAD, the doctors will complete a survey on self-reported learning, which contains a question on how many minutes they spent preparing for each case. The NOMAD team leader records technical difficulties, attendance, and timeliness for each case. Study data will be collected and managed using REDCap electronic data capture tools [42, 43] hosted at OPEN, Open Patient Explorative Network, OUH, Region of Southern Denmark.

### Statistical methods

This is a feasibility trial and statistical methods and analyses will be accordingly. Focus will be on assessing feasibility by reporting primary outcomes narratively. For the secondary outcomes measured before and after intervention, we will estimate confidence intervals (where appropriate) rather than hypothesis testing as suggested by Lancaster et al. [44]. We will assess acceptability of the questionnaires with completion rates. We will summarise the results using descriptive statistics, presenting categorical variables as numbers and percentages and continuate variables as medians with interquartile ranges.

### Sample size justification

In this study, sample size is a product of the number of NOMAD conferences and the number of patients referred. In order to assess feasibility of the intervention, we need study participants. We can only recruit study participants if doctors refer them. Getting doctors to refer patients require a change in their practice. Changing clinical practice requires time and dedicated effort. As many doctors have temporary employments (often 6–24 months), employer turnover is high in many hospital departments. We consider it necessary to collect data over at least 12 months, to allow the required change in practice to happen. Local circumstances allows us to collect data over 17 months, which means a possible 28 NOMAD conferences corresponding to a maximum of 112 patients. This sample size is bigger than median sample sizes in feasibility studies [45]. Skivington et al. argue that in order to increase the likelihood of an intervention being implemented, the context needs to be considered at an early stage [28]. Herein lies a recommendation of a thorough feasibility phase where contextual and organisational uncertainties are uncovered and adjusted for. A sample size calculation is not necessary as we are testing feasibility and not a hypothesis of effect [46].

### Dissemination

We will disseminate the results of this study with publication in peer-reviewed journals, oral presentations at relevant arenas, and in lay summaries to inform caregivers, patients, healthcare professionals, and researchers. Most importantly, it will inform the research group on how to proceed to an RCT, if the feasibility trial turns out positive.

### User involvement

We consider user involvement in the process of developing and testing interventions very important. Throughout the intervention development process, we have consulted both patients and intervention providers. Specifically, we completed interviews with patients early in the process in order to gain fundamental patient perspectives useful to the development process. In the development of the PRO questionnaire, users were involved in testing it before use. This followed established procedures in SDCO for patient and public involvement. Several of the doctors participating in the NOMAD also participate in the research group, and their hands-on input to the process is of great value. Further, we established a specific NOMAD user panel consisting of people with diabetes and multimorbidity, their family members and relevant healthcare professionals. The user panel will follow the NOMAD intervention as an advisory board, and we will invite them to discuss findings and data analyses. Furthermore, the user panel will be involved in the dissemination of the study.

## Discussion

This study will provide helpful information on the feasibility of MDTs for people with diabetes and multimorbidity as a clinical initiative. It will enlighten the practical aspects, the acceptability among intervention providers and recipients, and possible effects on HRQoL and organ-specific parameters. Moreover, it will orchestrate a future RCT aimed at testing effects of the NOMAD.

Multiple challenges relate to multimorbidity management. The high degree of complexity and heterogeneity in patients is not only a product of interactions between different conditions and treatments [5] but also personal, organisational, and societal dimensions added to the complexity [47–49]. How do we approach such a multiverse of challenges all interconnected and -dependent? A WHO report points out polypharmacy, complex care needs, and frequent and complex interactions with healthcare services as some of the aspects contributing to patient safety issues [1]. Designing initiatives addressing these risk factors seems obvious, although resource- and time-demanding.

This study has several potential limitations. As all participants receive the intervention, we cannot test the process of randomisation before a subsequent RCT. Another limitation is that the study collects and assesses a large variety of data. This can complicate the process and give rise to more practical issues compared to feasibility studies with fewer variables. Thirdly, the NOMAD team consists of doctors only, and lack of important capacities from other professions can be a limitation when working with multimorbidity care.

A strength of this study is a large sample size compared to most feasibility studies [45]. This will allow assessment of possible effect outcomes to a larger extent, compared to a feasibility study with a smaller sample size. The long inclusion time (17 months) will allow the intervention to settle in clinical practice in a way that we think can increase feasibility and acceptability to users. Hence, the likelihood of a successfully conducted future RCT increases. A third advantage is the use of PROs that aid clinical decision-making and secure a patient-centred approach, which we consider a key component to successful management of complex multimorbid patients.

Overall, this study will contribute with valuable knowledge on how to manage the complex challenges in the care of diabetes and multimorbidity and prepare the design of an RCT testing effect of the intervention.

### Supplementary Information

Additional file 1: Appendix 1: TIDieR template with intervention description.pdf. Condensed description of the intervention. Detailed Danish guideline is available upon request from the corresponding author.

Additional file 2: Appendix 2: Process indicators.pdf. A list of pre-defined elements that indicate good process.

Additional file 3: Appendix 3: Data collection.pdf. An overview of data collection over the course of the intervention and a list of variables. Figure 3a and 3b.

Additional file 4: Appendix 4: Journal audit and tool for registering NOMAD recommendations.pdf. A review of the first 31 patients to receive the intervention was performed pre-trial. This resulted in a system by which the NOMAD recommendations are to be registered.

## Data Availability

The datasets used and/or analysed during the current study will be available from the corresponding author on reasonable request.

## Abbreviations

NOMAD: Network of doctors for multimorbidity and diabetes
WHO: World Health Organization
MDT: Multidisciplinary team conference
SDCO: Steno Diabetes Centre Odense
RCT: Randomised controlled trial
PRO: Patient-reported outcome
HRQoL: Health-related quality of life
MRC: Medical Research Council
GP: General practitioner
HbA1c: Glycated haemoglobin type A1c
