# Supplementary material for: Network of doctors for multimorbidity and diabetes — the NOMAD intervention: protocol for feasibility trial of multidisciplinary team conferences for people with diabetes and multimorbidity
Source: Pilot Feasibility Stud. 2024 Jun 15;10:91. doi: 10.1186/s40814-024-01517-0 (PMC11179232; doi:10.1186/s40814-024-01517-0)
Supplement: Supplementary file 1 — Additional file 1: Appendix 1: TIDieR template with intervention description.pdf. Condensed description of the intervention. Detailed Danish guideline is available upon request from the corresponding author. [file 40814_2024_1517_MOESM1_ESM.pdf]

TIDieR

Multidisciplinary team conferences for people with diabetes and multimorbidity

Why:

A multidisciplinary team (MDT) approach for people with diabetes and multimorbidity may increase coordination and coherence in treatment which may lead to improved quality of life and possibly better health outcomes.
Many people with multimorbidity struggle with high treatment burden. Keeping up with clinical check-ups, hospital appointments at different departments, collecting and taking prescription medicine and living with physical disabilities and/or pain makes it hard work to live with multimorbidity. This can lead to mental exhaustion, loss of autonomy and severely impact quality of life. Instead of a disease-centered system where the patient travels between hospital departments and across sectors, we propose a patient-centered intervention, where the medical specialists gather around the patient.

What (material):

Material used in the intervention includes both material aimed at (1) the intervention providers (physicians), (2) the administrative staff (secretary) in relation to the intervention and (3) the intervention recipients (patients).
1) We made a document describing the MDT concept, who can refer patients, referral criteria, and how to refer. This was made available in the local collection of hospital instructions at Odense University Hospital. A separate info-package was made for the general practitioner. This package gives information on what MDT is, who can be referred and how.
2) We formed a separate instruction document for the administrative tasks, such as how to book patient in electronic booking system.
3) We made a pamphlet aimed at both patients and physicians. The pamphlet has two parts. One for the patient stating what the MDT is and how it works. The other part is for the physician. This provides a short description of referral criteria, how to inform the patient and how to refer.
The patients also receive a letter in their digital mailbox (E-boks) when they have stated that they want to participate in the MDT. This letter describes the MDT, when and where to meet up and what they can expect.

What (procedures):

Through regular MDT meetings and with a broad team in internal medicine, we hope to improve treatment and care for people with diabetes and multimorbidity. We have MDT meetings every other Thursday at 1-3 pm. The MDT can have 4 patient cases per meeting. To inform the MDT and put patient perspectives in focus, we send out a questionnaire to patients before the MDT meeting. Topics addressed in the questionnaire are health related quality of life, physical and mental well-being, diabetes distress, symptom burden, treatment burden and experienced involvement. Patients are also asked an open-end question about what is important to them. 6 months after the MDT meeting, the patient receives the same questionnaire once again. The answers are reviewed by healthcare professional. The patients are offered a telephone consultation to follow up on the past 6 months. How did it go? Did the MDT recommendations change anything for the patient?

Who provided:

The MDT team consists of diabetologist, cardiologist, pulmonologist, nephrologist and pharmacologist. They meet every two weeks and has a time slot of 30 minutes for each patient case. Patients can be referred from either hospital department or general practitioner (GP). GP is encouraged to participate in the MDT meeting through video conference. The patient is also invited to participate in the MDT meeting. Patients receive a questionnaire concerning physical and mental well-being, health related quality of life, diabetes distress, symptom burden, treatment burden and patient involvement. This constitute the patient-reported outcomes (PRO) element and is incorporated as the patients view in the MDT discussion.

Multidisciplinary team conferences for people with diabetes and multimorbidity

**Where:**	The intervention takes place at Steno Diabetes Center Odense, Odense University Hospital, Odense, Denmark. This is a public hospital, with a catchment area of 1.2 million people. The intervention is made available for all patients who meet referral criteria in this area.
**When and how much:**	The intervention period started January 2023 and will end December 2024. Patients can be referred several times to the MDT, if relevant. MDT meetings are held every other Thursday throughout the intervention period, excluding holidays.
**Tailoring:**	The intervention can be tailored to limited extent. Patients are invited to participate in their MDT discussion. They can be physically present at the meeting with the physicians, or they can opt for video conference. Not all patients are capable of answering questionnaire digitally, why some are exempt. Instead, the physician will try to obtain equivalent information by talking to the patient. Finally, external specialists can be invited to participate in certain cases. For instance, a specialist in palliative care can offer expertise in relevant cases.
