# Supplementary material for: Network of doctors for multimorbidity and diabetes — the NOMAD intervention: protocol for feasibility trial of multidisciplinary team conferences for people with diabetes and multimorbidity
Source: Pilot Feasibility Stud. 2024 Jun 15;10:91. doi: 10.1186/s40814-024-01517-0 (PMC11179232; doi:10.1186/s40814-024-01517-0)
Supplement: Supplementary file 2 — Additional file 2: Appendix 2: Process indicators.pdf. A list of pre-defined elements that indicate good process. [file 40814_2024_1517_MOESM2_ESM.pdf]

Appendix 2: Process indicators – elements that indicate good process

Recruitment criteria are met

Referral note is correctly/sufficiently filled out

Referral note is available for NOMAD team on time

PRO questionnaire is sent out on time

Patients answer PRO

The electronic questionnaire App is working properly

PRO answers are electronically available for NOMAD team on time

Patients are booked on NOMAD-list correctly (in electronic patient booking system, EPJ)

NOMAD team is sufficiently prepared

Prescription medicine review is conducted

Prescription medicine review available for NOMAD team on time

Preparation time for NOMAD team members is not exceeding time provided according to agreement

There are no technical issues during NOMAD regarding video, sound, Wi-Fi etc.

NOMAD team member are able to meet on time

NOMAD starts on time

NOMAD conference room is properly prepared according to guidelines (“Manual”, supplementary file)

Case discussions are orderly conducted

All medical specialties contribute with their speciality-specific assessment

There is clarity in who is in charge of writing NOMAD conclusion note

NOMAD conclusion note is written on site

NOMAD conclusion note is clear and concise, with recommendations provided in prioritized order

NOMAD conclusion note is forwarded correctly to GP/ correctly posted in electronic patient record

There is clarity in who is in charge of following up with the patient after NOMAD

Patients are informed of NOMAD conclusions/recommendations within 4 weeks after NOMAD

NOMAD recommendations are followed/implemented

Patients receive PRO2 questionnaire on time

Patients answer PRO2

NOMAD staff review PRO2 answers

Patients are correctly booked for phone-call

Patients receive phone-call 6 months after NOMAD

6-months phone-call is properly documented in electronic patient record along with PRO2 answers

In case of alarming PRO2 answers or other adverse aspects, proper action is taken
