# Supplementary material for: Network of doctors for multimorbidity and diabetes — the NOMAD intervention: protocol for feasibility trial of multidisciplinary team conferences for people with diabetes and multimorbidity
Source: Pilot Feasibility Stud. 2024 Jun 15;10:91. doi: 10.1186/s40814-024-01517-0 (PMC11179232; doi:10.1186/s40814-024-01517-0)
Supplement: Supplementary file 3 — Additional file 3: Appendix 3: Data collection.pdf. An overview of data collection over the course of the intervention and a list of variables. Figure 3a and 3b. [file 40814_2024_1517_MOESM3_ESM.pdf]

Appendix 3: Data collection

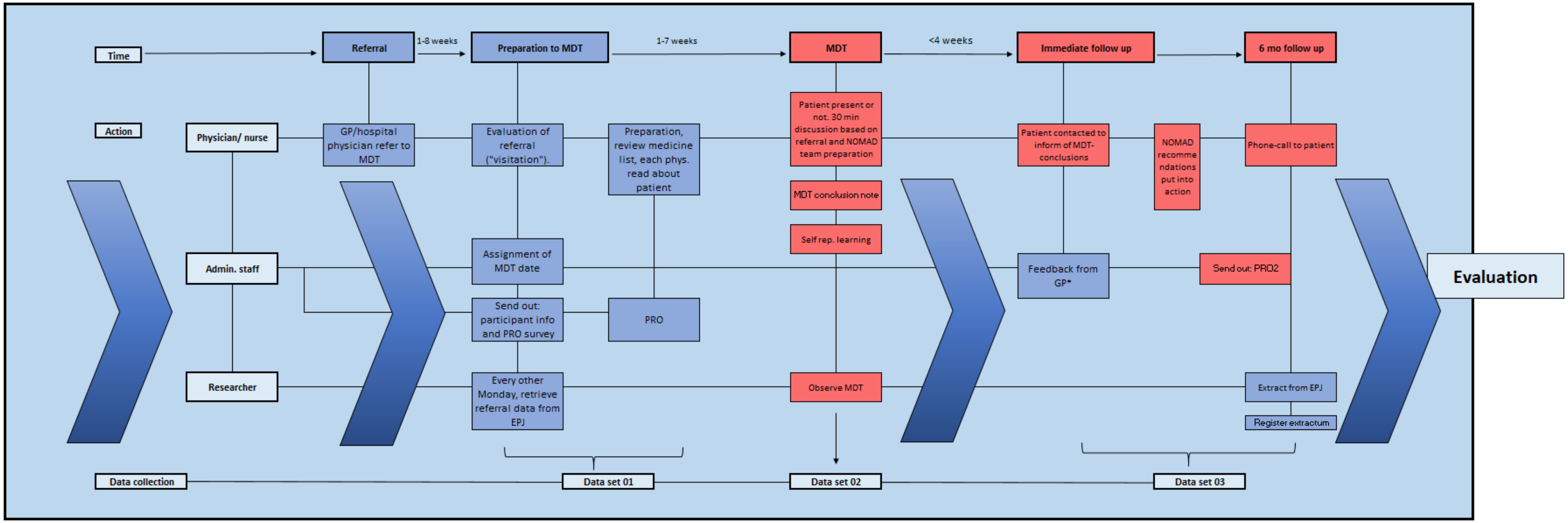

Figure 3a. Data collection timeline visualizing the data collection that will take place for each patient. Time is indicated at the top. The middle part describes different milestones in the NOMAD intervention. MDT: multidisciplinary team conferences. GP: general practice/practitioner. EPJ: electronic patient journal/records. Admin.: administrative. Self rep.: self reported. PRO: Patient reported outcome questionnaire. PRO2: second send out of PRO.

Data set 01

1.001 Who is referring
1.002 Reasons for referring

1.004 Sex
1.005 Age
1.006 DM type
1.007 DM debut
108 DM follow where
109 DM status
110 No of diagnoses
111 Comorbidities

115 HbA1C
116 LDL
117 Triglycerides
118 Creatinine
119 eGFR
120 UACR
121 Blood pressure

122 MTBQ score
123 EORTC-score
124 WHO-5 score
125 MDI2 score
126 ASS-2 score
127 SF12 answer
128 PAID-5 score
129 Patient involvement
130 EQ-5D-5L
131 Physician preparation tim
132 No. of health contacts last 12 months
133 No. of active trajectories
134 No. of medications

Data set 02

2001 Who is present
2002 Technical issues
2003 Patient present

2004 MDT conclusion note
2005 Schedule compliance

2010
2011
2012 NOMAD team learning survey
2013
2014

Data set 03

3001 Patient contact when
3002 Patient contact how

3003 No. of NOMAD recommendations put into action

308 DM follow where
309 DM status
310 No of diagnoses
311 Comorbidities

315 HbA1C
316 LDL
317 Triglycerides
318 Creatinine
319 eGFR
320 UACR
321 Blood pressure

322 MTBQ score
323 EORTC-score
324 WHO-5 score
325 MDI2 score
326 ASS-2 score
327 SF12 answer
328 PAID-5 score
329 Patient involvement
330 EQ-5D-5L

334 No of medications

Figure 3b. Overview of variables collected during the NOMAD intervention. Numbers are codes used for registration. Empty codes 2010, 2011, 2013 and 2014 are un-named items in NOMAD team learning survey.
