# Supplementary material for: Network of doctors for multimorbidity and diabetes — the NOMAD intervention: protocol for feasibility trial of multidisciplinary team conferences for people with diabetes and multimorbidity
Source: Pilot Feasibility Stud. 2024 Jun 15;10:91. doi: 10.1186/s40814-024-01517-0 (PMC11179232; doi:10.1186/s40814-024-01517-0)
Supplement: Supplementary file 4 — Additional file 4: Appendix 4: Journal audit and tool for registering NOMAD recommendations.pdf. A review of the first 31 patients to receive the intervention was performed pre-trial. This resulted in a system by which the NOMAD recommendations are to be registered. [file 40814_2024_1517_MOESM4_ESM.pdf]

Appendix 4 – Journal audit and tool for registering NOMAD recommendations

In 2021, 31 patients were discussed on the NOMAD conference. We conducted a journal audit on these 31 patients. Approval from the local department for quality studies in Odense University hospital was secured. One researcher read all 31 patient records. For all patients, the electronic records was studied from 3 months before the conference to 3 months after in order to gain information about hospital contacts, outpatient contacts, diagnoses, diabetes type, debut and status, number of medications. From the referral note, information like reason for referral and patient perspectives was noted. From the conferences conclusion note, key points of the discussions and conference conclusion were noted. In the time after the conference, information about when and how follow up was carried out was noted. All data were collected and organised in Excel 2016. All the different referral reasons where grouped in to apparent categories e.g. “symptoms related problem” or “medications related problem” and counted. The same strategy was used on the conference discussions/conclusion. Here the inductive process yielded many more categories, as the complexity increased. For instance, when the NOMAD recommended alterations in medication, it could fall into either one of seven categories: add, remove, increase, decrease, substitute, change way of administration or taper. The recommendations where divided into 5 groups: Medications, coordination, clinical issues, primary care and miscellaneous. The tool – a table of the different categories and actions to be counted per conference case – is provided below. With an aim to clarify what the NOMAD recommends and thus assess intervention impact, the NOMAD conclusion notes will be reviewed and registered using the tool below.

Categories	Actions													
**Medications**	Add	No.	Remove	No.	Increase	No.	Decrease	No.	Substitute	No.	Other adm.	No.	Taper	No.
**Coordination**	Treatment plan	No.	Confirm current treatment	No.	Plan for investigation	No.	Refer to examintation	No.	Appoint HCP in charge	No.				
**Clincal issue**	Assessment	No.	Tentative diagnosis	No.	Need more info from patient	No.								
**Primary care**	Follow up	No.	Examination	No.	Community health service	No.	Community social service	No.	Need more info from patient	No.				
**Miscellaneous**	Specified:													

Tool for registering NOMAD recommendations.
